# Multiple Sulfatase Deficiency (MSD): Review of the Literature and Case Reports of Two Siblings with Dental Caries and Trauma

**DOI:** 10.1155/2021/6611548

**Published:** 2021-02-16

**Authors:** Lorna Hirst, Gehan Abou-Ameira, Mari-Liis Uudelepp

**Affiliations:** Great Ormond Street Hospital, London, UK

## Abstract

Multiple sulfatase deficiency (MSD) (MIM # 272200) is an extraordinarily rare inborn error of metabolism (IEM). The phenotypic spectrum is largely heterogeneous and attributed to the combined effects of deficiencies in the nine sulfatases currently known to be related to human diseases. Systemic sequelae of MSD are vast and multisystemic, primarily encompassing developmental delay and neurological, cardiopulmonary, dermatological, gastroenterological, and skeletal manifestations. The dental phenotype is scarcely described in the literature due to a paucity of cases. Dental treatment under local and general anaesthesia mandates an integrated multidisciplinary approach to safeguard systemic health and optimise outcomes. This paper presents two siblings with multiple sulfatase deficiency who presented to the Paediatric Dental Department at Great Ormond Street Hospital, requiring comprehensive care under general anaesthesia for dental caries and trauma.

## 1. Introduction

Multiple sulfatase deficiency (MSD) (MIM # 272200) describes an exceptionally rare autosomal recessive inborn error of metabolism (IEM). Genetically, it is caused by a mutation in the sulfatase modifying factor 1 gene (SUMF1) [1, 2]. SUMF1 is a prerequisite for functioning sulfatases. Consequently, mutation in SUMF1 results in tissue accumulation of sulfatides, sphingolipids, sulphated glycosaminoglycans, and steroid sulphates [3]. Out of the seventeen known human sulfatases, nine are individually implicated in single sulfatase deficiency diseases [4, 5]. The clinical presentation of multiple sulfatase deficiency is therefore heterogeneous and attributed to the overarching features of the nine defined single sulfatase deficiencies. Subsequently, overarching features of various types of lysosomal storage diseases (LSD) are evident, such as metachromatic leukodystrophy (MLD), Maroteaux–Lamy syndrome, Hunter syndrome, Sanfilippo A syndrome, Morquio syndrome, and X-linked ichthyosis [4] (Table 1).

Genetically, more than thirty different mutations in the SUMF1 gene, which is located on chromosome 3p26.1, have been discovered [1, 2, 6, 7]. SUMF1 encodes formylglycine-generating enzyme (FGE), which is essential for the posttranslational activation of all sulfatases in the endoplasmic reticulum [4, 6]. The severity of FGE protein instability and residual catalytic ability influence the clinical severity and phenotype of MSD [6, 7].

## 2. Cases

This article presents the case of two male siblings at six and eleven years of age who were treated at the paediatric dental department of a tertiary referral hospital for comprehensive caries and dental trauma management. Both siblings have genetically confirmed MSD with homozygous mutations c.836 C > T, p.Ala279Val in the SUMF1 gene. The brothers' parents are second cousins. Their older brother died shortly after birth due to renal agenesis.

Case one presents the older sibling, an eleven-year-old male. He was delivered vaginally following an unremarkable gestation period. In the first few months, he had formidable feeding difficulties and recurrent chest infections requiring hospitalisation. His height, weight, and head circumference were all persistently below growth centiles. He presented with slow psychomotor development from early age but was able to walk from two and a half years of age and speak a few words by three years of age. He was diagnosed with MSD at four years of age and subsequently started to show first signs of regression of his skills from five-six years of age. He also presented with coarse facial features, postretinal visual dysfunction, myopic astigmatism, epilepsy, and ichthyosis. His medications included macrogol, lamotrigine, melatonin, and salbutamol.

He was referred to the dental department internally from his metabolic team aged eleven. On examination, hypoplasia of all first and second premolars and first permanent molars was evident. Soft tissues were normal, and no caries were detected.

**Figure 1:**
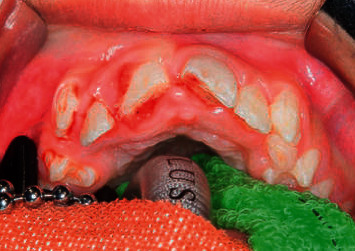
Initial presentation of dental trauma showing a 3 mm intrusion of the UR1 and a complicated mesial incisal fracture of the UL1.

**Figure 2:**
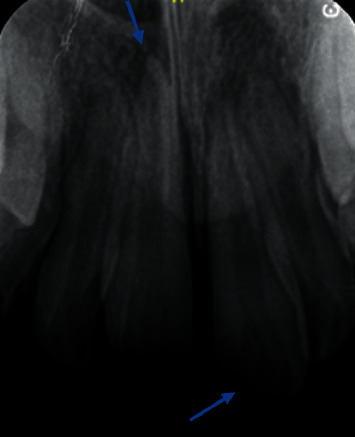
Intrusion injury of the UR1 (left arrow) and a complicated mesial incisal fracture of the UL1 (right arrow).

**Figure 3:**
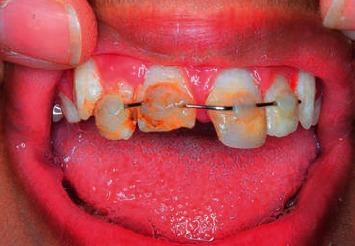
Splinting with a nonactive orthodontic wire and composite after surgical repositioning.

Six months later, he presented to the dental department with trauma of the maxillary right and left central incisors (UR1 and UL1) (Figures 1 and 2). Extraorally, oedema and erythema of both lips were observed. Intraorally, a small laceration on the labial mucosa adjacent to the mandibular left lateral incisor (LL2) was evident. Dental injuries included a mesial incisal complicated fracture of the UL1 with grade 2 mobility and a 3 mm intrusion of the UR1. Maxillary occlusal radiography identified a potential tooth fragment in the upper lip. Challenging cooperation necessitated management under general anaesthesia. Stage one root canal treatment (RCT) of the UR1, UL1, surgical repositioning of UR1, and splinting (Figure 3) was completed under general anaesthesia. Two further general anaesthetics were required for second stage RCT, treatment of persistent endodontic infection, composite build-up of the UL1, and removal of the splint (Table 2). Clinical and radiographic follow-up will be necessary to rule out apical root resorption, ankylosis, and endodontic failure.

Case two presents the younger brother, aged six. He received a confirmed diagnosis of MSD in his first months of life. Additionally, he was also antenatally diagnosed with thoracic open myelomeningocele and hydrocephalus, which required immediate neurosurgical correction postnatally. He was born from normal vaginal delivery with bilateral talipes, which was treated conservatively.

At age six, developmental delay is evident. His motor function is complicated by paraplegia and has resulted in him being wheelchair bound. He additionally presents with coarse facial features, Arnold–Chiari syndrome, sensorineural hearing loss, lumbar kyphosis, ichthyosis, short stature, recurrent chest infections, and neuropathic bladder. His medications include macrogol, salbutamol, urea cream, multivitamins, and a high fibre nutritionally complete supplement (7.4 g sugar/100 ml) three times daily.

**Figure 4:**
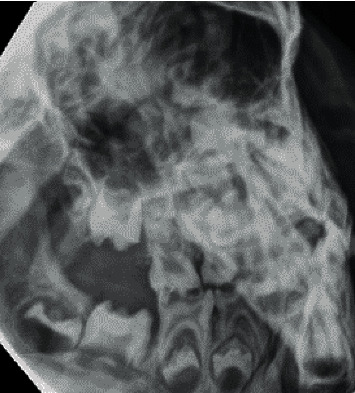
Gross caries experience aged 6.

He was internally referred to the dental department for odontogenic pain at six years of age. On intraoral examination, hypoplasia of all primary molars was noted in addition to extensive caries (14 primary teeth) (Figure 4). Preformed metal crowns were contraindicated due to a high frequency of MRI scans. Arrangements were made under general anaesthesia for their extraction along with the mobile maxillary primary canines (ULC and URC) (Table 2).

## 3. Discussion

Multiple sulfatase deficiency (MSD) describes an extraordinarily rare inborn error of metabolism, with a prevalence of <1 in 1 million live births [2]. The pathophysiology of MSD explains the clinical heterogeneity with both siblings displaying symptomology across the range of sulfatase deficiency syndromes, selected mucopolysaccharidoses, metachromatic leukodystrophy, X-linked ichthyosis, and X-linked chondrodysplasia punctata.

A paucity of cases describing dental management, in conjunction with phenotypical diversity and complex multisystemic involvement, mandate specialist multidisciplinary care. Multifaceted behavioural difficulties, as seen in the constituent sulfatase-deficient mucopolysaccharidosis (IID, II, and IIIA), complicate or preclude dental treatment under local anaesthesia, necessitating general anaesthesia (GA) in many cases. Dental treatment planning should therefore concentrate on achieving long-term restorative success and potentially more radical treatment approaches, such as extracting nonvital primary molars rather than pulp therapy to mitigate the risks of sequential general anaesthetics.

Systemic sequelae magnify risk, as evidenced by the American Society of Anaesthesiologists Grade III (ASA III) assigned to both cases. Risks become further tangible when multiple GAs are required, such as in case 1. Anaesthetic challenges mirroring MPS are evident, such as glycosaminoglycan accumulation in the head and neck region, leading to difficulties in intubation in conjunction with potential craniocervical instability and other skeletal abnormalities, cardiac comorbidities, restrictive lung disease, hydrocephalus, and seizures [4, 8]. Anaesthetists familiar in the management of IEMs, particularly lysosomal storage disorders, are therefore invaluable.

Dentally, both siblings showed enamel hypoplasia, as seen in Morquio A syndrome (MPS IVa) [9], and deficiency in galactosamine-(*N*-acetyl)-6-sulfate sulfatase (GALNS) [10]. A high caries experience was evident in case 2. This is likely attributable to nutritional supplements, high in carbohydrates, that is, sugar (7.4 g sugar/100 ml), given to support optimal nutrient intake due to feeding difficulties. Evidently, this predisposes to dental caries and necessitates implementation of an aggressive caries preventive regime to offset elevated anaesthetic risk.

The low prevalence and high heterogeneity of MSD contribute to challenges and delayed diagnosis in many cases [1]. Scarcity of knowledge of all seventeen known sulfatase enzymes means that delineation of the complete phenotypical spectrum of MSD has unlikely been described. The amalgamation of multiple sulfatase deficiencies concurrently may present with novel pathophysiological effects not identified from single sulfatase deficiencies. Dental manifestations are scarcely described in the literature, emphasizing the importance of ameliorating knowledge in order to understand MSD complications and formulate dental management protocols. MSD remains an untreatable IEM, with a guarded prognosis [5, 6]. Dental management should therefore focus on optimising preventative strategies, alleviating dental pain and treatment planning for long-term restorative success.

The utility of the multidisciplinary team cannot be understated in coordinating the holistic management of MSD patients. Extensive multisystemic sequelae have the propensity to complicate management under both local and general anaesthetic. Paediatric dentists should be aware of the material anaesthetic risk and understand the importance of coordinating management with the patients treating medical team.

## Figures and Tables

**Table 1:** Clinical disorders attributed to individual sulfatase deficiencies [3, 7, 10].

Deficiency	Condition	Common clinical features
Arylsulfatase A	Metachromatic leukodystrophy	Leukodystrophy, central and peripheral demyelination, neuropathy, progressive spasticity and regression of skills, optic atrophy

Arylsulfatase B	Mucopolysaccaridosis type VI (MPS VI, Maroteaux–Lamy syndrome)	Skeletal dysplasia, short stature, degenerative joint disease, cardiac valve disease, hearing loss, obstructive sleep apnoea, corneal clouding, carpal tunnel disease, umbilical and inguinal hernia, coarse facial features, normal intellect

Arylsulfatase C	X-linked ichthyosis	Excessive skin scaling and hyperkeratosis; may be associated with developmental delay, autism, and attention deficit hyperactivity disorder

Arylsulfatase E	X-linked recessive chondrodysplasia punctata	Abnormal cartilage and bone development, short stature, tracheal stenosis, spinal cord compression, developmental delay, hearing loss, visual disturbances, heart defects, distinctive facial features

Galactosamine (*N*-acetyl)-6-sulfate sulfatase (GALNS)	Mucopolysaccaridiosis type IVA (MPS IVA, Morquio A syndrome)	Very severe skeletal dysplasia, including hip dysplasia, genu valgum, severely poor growth and odontoid dysplasia with high risk for atlanto-occipital subluxation, hepatomegaly, coarse facial features, normal intellect

*N*-Acetylglucosamine-6-sulfatase	Mucopolysaccharidosis type IIID (MPS IIID, Sanfilippo syndrome type IIID)	Intellectual disability with progressive regression of skills, severe behavioural difficulties, seizures, organomegaly, corneal clouding, hearing loss, hepatomegaly, coarse facial features

Iduronate-2-sulfatase (IDS)	Mucopolysaccharidosis type II (MPS II, Hunter syndrome)	Severe intellectual disability, behavioural difficulties, seizures, skeletal dysplasia, corneal clouding, cardiopulmonary disease, organomegaly, coarse facial features

Heparin sulfate sulfatase	Mucopolysaccharidosis type IIIA (MPS IIIA, Sanfilippo syndrome type IIIA)	Severe intellectual disability with progressive regression of skills, severe behavioural difficulties, seizures, organomegaly, corneal clouding, hearing loss, hepatomegaly, coarse facial features

**Table 2:** Metabolic, medical, and dental diagnoses and management of the two siblings.

Patient demographics	Metabolic diagnosis and management	Medical diagnoses and management	Dental management
*11-year-old male*	Multiple sulfatase deficiency (homozygous mutations c.836 C > T, p.Ala279Val in SUMF1 gene)	(i) Epilepsy	*Dental diagnoses*
		(ii) Recurrent chest infections	(i) Enamel hypoplasia
		(iii) Global developmental delay	(ii) Complicated fracture UL1; grade 2 mobility-3 mm intrusion UR1
		(iv) Postretinal dysfunction	
		(v) Myopic astigmatism	
		(vi) Ichthyosis	*Dental management*
		*Medication*	*Fissure sealants*: UR6 UL6 LR6 LL6
		(i) Macrogol	*Root canal treatment*: UR1, UL1
		(ii) Lamotrigine	*Surgical repositioning*: UR1
		(iii) Melatonin	*Explore maxillary lip*
		(iv) Salbutamol	*Composite build-up*: UL1 *extraction*: ULC (mobile)

*6-year-old male*	Multiple sulfatase deficiency (homozygous mutations c.836 C > T, p.Ala279Val in SUMF1 gene)	(i) Arnold–Chiari syndrome	*Dental diagnoses*: caries and hypoplasia *Extractions*: UREDCBA ULABCDE LREDC LLCDE Fluoride varnish 22,600 ppm
		(ii) Thoracic open myelomeningocele	
		(iii) Hydrocephalus	
		(iv) Sensorineural hearing loss	
		(v) Paraplegia	
		(vi) Lumbar kyphosis	
		(vii) Ichthyosis	
		(viii) Bilateral talipes	
		(ix) Global developmental delay	
		(x) Short stature	
		(xi) Recurrent chest infections	
	
		*Medications*	
		(i) Macrogol	
		(ii) Salbutamol	
		(iii) Urea cream (10%)	
		(iv) Multivitamins	
		(v) Nutritionally complete food supplements	

## Data Availability

No data were used to support the findings of this study.
